# Summer drought impacts micropredator abundance and microbial food web structure in a rewetted fen peatland

**DOI:** 10.1093/ismeco/ycag169

**Published:** 2026-06-16

**Authors:** Die Hu, Haitao Wang, Verena Groß, Anna Burns, Tim Urich

**Affiliations:** Institute of Microbiology, University of Greifswald, 17489 Greifswald, Germany; Institute of Microbiology, University of Greifswald, 17489 Greifswald, Germany; Grassland and Forage Sciences, University of Rostock, 18059 Rostock, Germany; Institute of Microbiology, University of Greifswald, 17489 Greifswald, Germany; Institute of Microbiology, University of Greifswald, 17489 Greifswald, Germany; Institute of Microbiology, University of Greifswald, 17489 Greifswald, Germany

**Keywords:** soil bacterivores, metatranscriptomics, bacterivorous bacteria, *Ca*. Patescibacteria, bacteriophage

## Abstract

Summer drought significantly affects soil microbiome diversity and functioning, and metabolic interactions in wetland ecosystems, yet its effects on the microbial food web are less understood. We investigated the dynamics of bacterivorous microorganisms and prey bacteria (PBac) in a rewetted fen peatland before, during, and after a summer drought using small subunit ribosomal RNA (SSU rRNA) gene sequencing and quantitative metatranscriptomics. We identified bacterivores including bacteria (e.g. Myxobacteria), protists, and nematodes, as well as *Ca*. Patescibacteria and *Diapherotrites*, *Parvarchaeota*, *Aenigmarchaeota*, *Nanoarchaeota,* and *Nanohaloarchaeota* archaea (PD) having a host-dependent lifestyle. In addition, bacteriophage transcripts were enumerated. The summer drought increased the diversity of bacterivorous protists (BPro) and bacteria (BBac) in the fen, along with decreases in the water level and increases in redox potential. The SSU rRNA transcripts abundance for all bacterivores (with the exception of the PD) increased during the drought, reaching a peak in October. The transcript abundance of all bacterivores remained above predrought level for at least four months after the drought ended. This became more evident when assessing the bacterivore-to-prey-bacteria ratios, with the ratios of aerobic BBac, BPro, and nematode to PBac increasing by more than two-fold compared to predrought. In contrast, the PD and bacteriophage-to-prey ratios remained rather stable. This study provides a holistic view of the diversity and composition of bacterivores in a fen peat microbiome, including the PD and bacteriophages, and suggests a long-lasting impact of summer drought on bacterivore and food web dynamics. The strong responses of aerobic bacterivores may lead to changes in ecosystem functioning through modulated trophic interactions in a changing climate.

## Introduction

Soil bacterial communities are extremely diverse [1] and represent the dominant primary decomposers of organic substrates [2]. Biotic interactions with soil organisms, such as predators and parasites, influence the decomposer community structure, and organic matter cycling in the soil [3]. Bacterivores are organisms that primarily consume bacteria, assimilating part of the prey biomass into their own biomass, and contributing to carbon and nutrient mineralization through respiration and excretion.

**Figure 1:**
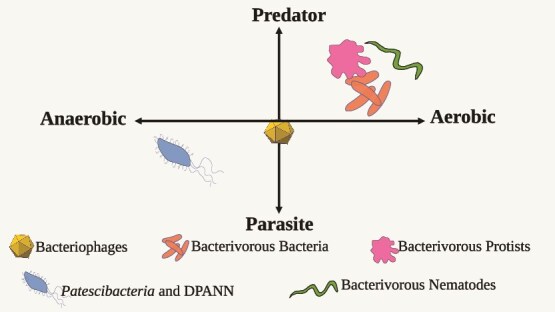
A coordinate system representing the trophic mode of soil (micro-)organisms and bacteriophages based on relationship to oxygen and trophic strategy. The term “predator” is defined as organisms that hunt, kill, or consume other organisms (prey) to obtain energy, usually acting quickly and independently. The term “parasite” is defined as organisms that live on or inside a host, feeding on it over time without immediately causing death. The known *Ca.* Patescibacteria and DPANN archaea (*Ca.* Diapherotrites, *Ca.* Parvarchaeota, *Ca.* Aenigmarchaeota, Nanoarchaeota, and *Ca.* Nanohaloarchaeota) have an anaerobic energy metabolism. The bacteriophages are non-living organisms that are not dependent on oxygen. The other three bacterivore groups are primarily classified as having an obligate aerobic energy metabolism.

Bacterivores span all three domains of life, including eukaryotes (protists and nematodes), bacteria, and archaea as well as bacteriophages [4–6]. In this study, we define both bacterial predators and parasites as “bacterivores” (Fig. 1). Due to the high diversity of bacterivorous taxa and their complex and specific interactions with their potential prey bacteria (PBac), the relationships between different bacterivorous groups and their respective prey are still poorly understood in the soil environment [7].

Bacterivorous bacteria (BBac), protists (BPro), and nematodes (BNem) are mostly aerobic organisms with a well-known predator strategy (Fig. 1) [8, 9]. BPro and BNem are among the most well-studied bacterivores in soils [10–12] and are considered to be the most important soil bacterivores. Within peatlands, they are known to be influenced by climate change factors, including warming and elevated carbon dioxide (CO_2_) levels treatment [13–15]. Well-known BPro belong to the *Amoebozoa*, *Cercozoa*, *Ciliophora*, *Foraminifera*, *Euglenozoa*, and *Heterolobosea* [5, 16], among others. BNem include orders, such as *Araeolaimida*, *Chromadorida*, *Desmodorida*, *Enoplida*, *Monhysterida*, *Rhabditida*, and *Triplonchida* [12]. Next to eukaryotic bacterivores, certain groups of bacteria, including Myxobacteria, *Bdellovibrio*, and like organisms (BALOs), *Lysobacter*, *Vampirovibrio, Cytophagia,* and *Streptomyces*, can show a predatory lifestyle [5, 17–19]. Myxobacteria SSU rRNA reads were reported in soils with even the highest proportion of other bacterivores [5]. BALOs and *Vampirovibrio* are obligate BBac that feed directly on living bacterial cells [20, 21]. Others, such as *Cytophagia*, *Streptomyces*, *Myxobacteria*, and *Lysobacter* are facultative BBac, meaning they can gain energy and nutrients from organic matter as well. Obligate BBac demonstrated faster growth and higher assimilation rates than facultative BBac [17].

Many studies suggest that viruses that infect bacteria (bacteriophages) also act as bacterivores by hijacking bacterial resources for their own replication [4, 22, 23]. Likewise, two large phylogenetically distinct groups, the bacterial candidate phyla radiation (CPR) and the archaeal DPANN (*Diapherotrites*, *Parvarchaeota*, *Aenigmarchaeota*, *Nanoarchaeota,* and *Nanohaloarchaeota*) superphylum, are thought to acquire carbon and essential nutrients from their hosts for growth [24, 25]. In many cases, the small genome of CPR and DPANN lacks genes encoding biosynthetic pathways, such as those for amino or nucleic acid synthesis [26]. Also the predicted energy metabolism is often fermentation, lacking complexes associated with both aerobic and anaerobic respiratory chains, respectively [27]. Many CPR taxa, including *Ca*. Microgenomates (OP11), *Ca*. Parcubacteria (OD1), *Ca*. Gracilibacteria (BD1–5), *Ca*. Saccharibacteria (TM7), and *Ca*. Dojkabacteria (WS6), have recently been reclassified under the superphylum *Ca*. Patescibacteria [28, 29]. *Ca*. Patescibacteria and DPANN archaea (PD) are often found in oxygen-limited or anaerobic environments [30]. Evidence for a “vampiristic” metabolism—meaning a dependence on other microbes for food and energy rather than producing them independently—was also found in DPANN [6]. These findings emphasize the need to consider the PD lineages with their host-dependent lifestyle as potential bacterivores based on current insights into their ecophysiology.

In recent decades, efforts to rewet formerly drained peatlands to restore biodiversity, carbon sink function, and mitigate climate change have increased worldwide [31, 32]. These rewetted peatlands are considered novel ecosystems because rewetting may take decades to return them to a natural state [33, 34]. Additionally, temperate rewetted fens are subject to more frequent extreme weather events, such as summer droughts, due to climate change. In 2018, Europe experienced a severe summer drought, causing changes in its methane-cycling microbiome composition of a rewetted fen and its associated functions [32, 35, 36]. The introduction of oxygen into the peat caused by a drought-driven water-table drop could impact the carbon storage function in rewetted peatlands by increasing organic carbon decomposition by aerobic organisms [32, 37]. Both the several-fold lower free energy yields of anaerobic pathways of C mineralization as well as the postulated “enzyme latch mechanism” are considered important for reducing microbial activity under anoxia enough to accommodate soil organic carbon (SOC) accumulation [38]. From a broader perspective, the introduction of oxygen during drought may thus benefit aerobic organisms, while inhibiting anaerobic microorganisms in the peatland. On the other hand, larger organisms like BNem and BPro might be more sensitive to environmental changes, such as temperature and oxygen, than smaller microorganisms like BBac and PD, meaning that a severe summer drought may exacerbate the mortality of BPro and BNem.

This study investigated if summer drought affected the trophic structure of the microbial foodweb in a rewetted fen. To this end, the long-term temporal dynamics of both prokaryotic and eukaryotic bacterivores was studied using amplicon sequences of the 16S and 18S rRNA genes. Additionally, we used quantitative metatranscriptomics to obtain a holistic overview of the temporal dynamics of all the above-mentioned bacterivorous (micro-)organisms and their response to a summer drought in the rewetted percolation fen [39–41]. Metatranscriptomics is currently the only method that can capture all the above-mentioned organisms in one experimental approach [42]. We hypothesize that (i) summer drought will increase the abundance of aerobic bacterivores, such as BBac, BPro, and BNem due to increased oxygen availability, (ii) that the abundance of bacteriophages is entirely dependent on bacteria will follow changes in PBac, and (iii) that the proportion among bacterivores of Patescibacteria and DPANN will drop during the drought due to their anaerobic and host-dependent lifestyle.

## Materials and methods

The samples were collected from a percolation rewetted fen from WETSCAPES project site [31]. A total of 36 samples were collected in triplicate from April 2017 to October 2019 at a depth of 5–10 cm below the soil surface. DNA-based 16S rRNA and 18S rRNA amplicon sequencing was conducted on 36 samples, while metatranscriptomic sequencing was conducted on 15 samples from April to October 2018 and in February 2019. The drought period, which occurred from June to October 2018, was characterized by a decline in water level, which remained below the sampling depth.

All raw amplicon sequence data were demultiplexed and processed using the dada2 (v1.8.0) pipeline as described in Wang *et al*. [43]. The final representative sequence of each amplicon sequence variant (ASV) was assigned to taxonomy against a modified version of the SILVA SSU ref NR 128 database using the lowest common ancestor (LCA) algorithm with MEGAN5 [44]. Potential bacterivores were assigned to four categories: BBac, *Ca*. Patescibacteria and DPANN archaea from the 16S rRNA, and BNem and BPro from the 18S rRNA (Table 1). We defined total non-predatory bacterial abundance as a proxy for PBac, as outlined by Petters *et al*. [5]. This simplification does not capture taxon-specific feeding preferences, but it provides a practical approach for assessing broad-scale trophic dynamics in complex soil communities.

**Table 1:** Overview of soil bacterivores across three domains of life and bacteriophages included in this study. BBac, BPro, and BNem were designated primarily based on [5]. Bacteriophages, which replicate using bacterial resources and lyse their hosts under certain conditions, are also considered bacterivores. In addition, *Ca.* Patescibacteria and DPANN archaea represent candidate bacterial and archaeal lineages with parasitic lifestyles using bacterial hosts in limited-oxygen environments.

**Groups**	**Taxonomy**	**Citation**
Bacterivorous bacteria (BBac)	All *Myxococcota* (formerly order *Myxococcales*), with the exception of genus *Sorangium*. Order *Cytophagales*, Order *Vampirovibrionales*, *Bdellovibrionota* (formerly *Bdellovibrionales*), with the exception of family *Oligoflexales*, Genus *Streptomyces*, Genus *Lysobacter*, and Genus *Daptobacter* were not found in this study	[5, 17, 75]
*Ca.* Patescibacteria and DPANN (PD)	Candidate Phyla Radiation (CPR, most are now superphylum *Ca.* Patescibacteria); archaea of the phyla *Diapherotrites, Parvarchaeota, Aenigmarchaeota, Nanoarchaeota*, and *Nanohaloarchaeota* (DPANN)	[76]
Bacterivorous Protists (BPro)	Superphylum *Amoebozoa*, Phylum *Cercozoa*, Phylum *Heterolobosea*, Phylum *Ciliophora* Phylum *Foraminifera*, and Phylum *Euglenozoa* were not found in this study	[5]
Bacterivorous Nematodes (BNem)	Orders *Araeolaimida*, *Chromadorida*, *Desmodorida*, *Enoplida*, *Monhysterida*, *Triplonchida*, *Rhabditida*	[5]
Bacteriophage	Family *Fiersviridae* (formerly family *Leviviridae*)	[65]

The processing of metatranscriptomic data was described in Wang *et al*. [35]. Briefly, the raw sequences were merged and categorized into small subunit (SSU) rRNA, large subunit rRNA and non-rRNA fractions [45]. To maintain consistency throughout the study, taxonomy was assigned with the same SILVA 128 database using the LCA algorithm (min score 155; max expected 0.01; top percent 4.0; min support 1) with MEGAN6 [46]. However, the latest taxonomic names from the new database have been adapted when necessary, e.g. *Ca*. Patescibacteria (which was CPR in the old database). Non-rRNA reads were aligned to the NC_nr database (accessed 12 April 2020) using DIAMOND [47], with taxonomies assigned in MEGAN5. On average, 3040 reads per sample corresponded to viral sequences, of which 42.7% were classified to bacteriophages in family *Leviviridae*. A total of 14 metatranscriptomic samples remained for our downstream analyses, excluding one due to strong divergence from the other two replicates.

Community diversity was assessed using the Shannon index using the R package *phyloseq* (v1.50.0) [48]. Differences between groups were tested with Kruskal–Wallis and Dunn’s *post hoc* tests. Community composition was analyzed using principal coordinates analysis (PCoA) based on Bray–Curtis distances after Hellinger transformation, and a PERMANOVA was used to assess temporal effects. Spearman correlations were examined relationships between bacterivores, PBac, and soil properties. *P*-values were false discovery rate (FDR)-adjusted, with significance at *P* < .05. The absolute abundance of SSU rRNA transcripts and viral RNA abundance were calculated based on total RNA content [32, 39, 40]. The calculation of the bacterivore-to-PBac ratio involved the ratio of transcript abundance of bacterivorous groups to that of PBac, scaled to 100, based on RNA transcripts per gram of dry weight (DW) soil. All steps are detailed in the Supplementary information.

## Results and discussion

We first investigated the long-term dynamics of different bacterivores in the peat-soil microbiome using 16S and 18S rRNA amplicon sequencing, which revealed temporal patterns in their diversity and responses to environmental fluctuations, especially during the 2018 summer drought. The subsequent application of quantitative metatranscriptomics enabled a comprehensive and holistic community perspective by quantifying the abundance of SSU rRNA across three domains of life, in addition to identifying RNA viruses [42]. The RNA-based analyses revealed distinct patterns of interactions between PBac and different bacterivores before, during, and after the summer drought.

### Summer drought affected oxygen- and nutrient-status in a rewetted fen

**Figure 2:**
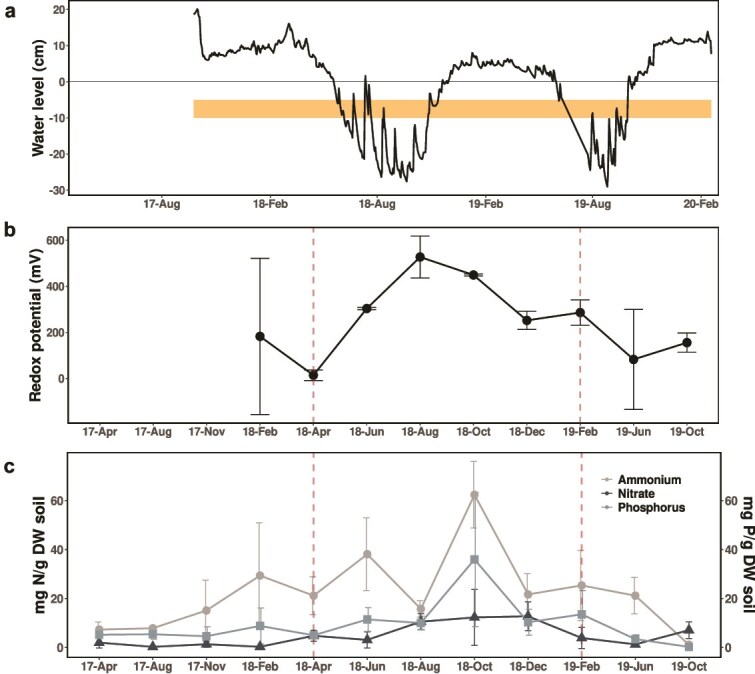
The dynamics of water level (a), redox potential (b), and the concentration of nutrients (c) from 2017 to 2019 in a rewetted fen. Error bars indicate standard errors (*n* = 3). The water level was scanned every minute and recorded at 15-min intervals. The horizontal bar in (a) indicates the sampling depth (5–10 cm). The two dashed vertical lines indicate the studying samples from April 2018 to October 2018 and February 2019, when further metatranscriptomic sequencing was performed.

The water level was recorded from September 2017 to October 2019 (Fig. 2a), with some timepoints also reported by Wang *et al*. [35, 49]. The water level remained relatively stable at approximately 10 cm above the soil surface from October 2017 to February 2018. From then on, a gradual decline was observed, with the water level falling below the sampling depth at 10 cm from June to October. The lowest water level was recorded in August 2018, reaching 21 cm below the surface, which corresponded to the driest month during the drought.

Water level was negatively correlated with redox potential at 10 cm below the surface (Spearman’s *ρ* = −0.80, *P* < .05). June, August, and October 2018 samples had a redox potential above +300 mV (Fig. 2b) and were therefore considered to be oxic systems with oxidative conditions [50], indicating a summer drought lasting until autumn over a period of five months. Drought events can compromise the suboxic or anoxic nature of peatlands belowground, promoting a shift towards oxic conditions that may undermine their carbon storage function and increase the risk of peat degradation and subsequent CO_2_ emissions even after rewetting [51]. It is well established that in the presence of oxygen, the most favorable electron acceptor, aerobic metabolism provides ample free energy for larger microbial communities [9, 14, 52]. This phenomenon is further substantiated here by the observed significantly positive correlation between the absolute SSU rRNA transcript abundance (per gram of DW soil) of PBac, BBac, BPro, and the redox potential (Supplementary Table S1). This suggests that the formation of an oxic environment may have benefitted the growth of aerobic PBac and aerobic bacterivores, such as BBac and BPro during the summer drought.

The concentration of nutrients increased during the summer drought (Fig. 2c). An increase in phosphorus from 2017 to 2018 and a significant decrease from 2018 to 2019 were observed (*post hoc* Kruskal-Wallis test, *P* < .05). However, pairwise comparisons using Dunn’s test with multiple-testing correction did not reveal significant differences in nitrate concentrations between specific month pairs (all adjusted *P* > .05). Burns *et al*. [53] demonstrated increased nitrates concentrations that may be supplied by plant–microbe and microbe–microbe interactions, as indicated by the functional genes and transcripts of microbes involved in nitrification.

### Temporal dynamics of microbiome diversity and composition

In total, 15 609 prokaryotic 16S rRNA gene ASVs and 4711 eukaryotic 18S rRNA gene ASVs were obtained from 36 samples across the time period and sorted into PBac as well as the different bacterivorous groups. We calculated Shannon diversity indices to quantify species diversity by accounting for both the number of species (richness) and the evenness of their distribution (Supplementary Fig. S1).

The observed trend of Shannon diversity among PBac was consistent over a period of 3 years (Supplementary Fig. S1). In general, the diversity of prokaryotic bacterivores, such as BBac and PD, was higher in 2018 than in 2017. There was a significantly greater diversity of BBac in 2018 compared to 2017 (*P* < .05, Supplementary Table S2). This may reflect temporal environmental changes (e.g. lower water levels, higher redox potential, and increased nutrient concentrations) and shifts in host bacterial communities, which can adapt more rapidly than eukaryotes due to their short generation times and large population sizes [54]. A decrease in the diversity of BBac and PD was observed in 2019, which may be due to a 2-month shorter drought period in 2019. This finding, nevertheless, may be influenced by incomplete data collection during the second drought period in that year. Consequently, the underlying causes of the observed decline remain uncertain.

The Shannon diversity of both BPro and BNem remained constant in 2018 and 2019. However, significant differences in BNem Shannon diversity were observed between spring and winter samples (*P* < .05, Supplementary Table S2). This finding indicates that the BNem may be more susceptible to seasonal variations, which is in line with the results of Wang *et al*. [43] and van den Hoogen *et al*. [55].

**Figure 3:**
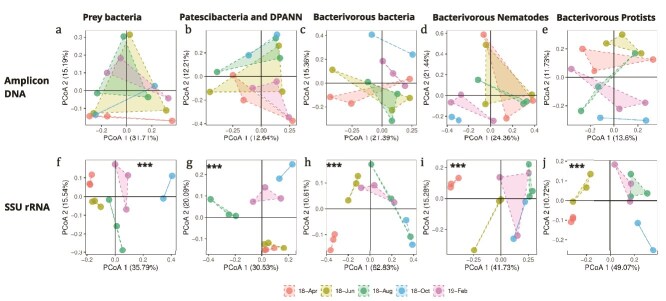
PCoA plots showing the dynamics in the soil community structure of PBac and different bacterivores over the drought cycle based on the amplicon sequences (a-e) and metatranscriptomic SSU rRNA sequences (f-j), respectively. *** indicated a significant difference (*P* < .001) between time points within each micro-(organisms) group based on the PERMANOVA analysis.

Despite that microbiome structures were not significantly different between months based on the DNA amplicon data, they were significantly different based on SSU rRNA as shown in the PCoA plots (Fig. 3), which is line in with an earlier finding of eukaryote dynamics [43]. Specifically, the SSU-rRNA-based microbiome profiles explained twice as much variation in the PCoA for prokaryotic bacterivores compared to the DNA amplicon datasets.

The rRNA data revealed that the bacterivorous groups in October, which corresponded to the end of the drought, exhibited the highest dissimilarity in comparison with those in April before the onset of the drought. However, the community composition did not return to its previous state after the drought ended, given that samples in February 2019 showed a high dissimilarity to the composition in April 2018 (Fig. 3). Spearman correlations (Supplementary Table S1) indicated that the water level and redox potential were both significantly associated with the transcript abundance of PBac as well as BBac and BPro. Therefore, the observed dissimilarity likely reflects hydrological differences, including changes in the water level and the associated shift between anoxic and oxic conditions during drought versus non-drought periods. Moreover, the role of runoff-driven dispersal should be considered but remains uncertain, as we did not directly measure the movement or dispersal of bacterivores.

### Increase of aerobic bacterivore transcript abundance during drought

RNA, specifically ribosomal RNA content (per gram soil or peat) of soil microbiomes, has been used as a proxy for microbial biomass per gram soil or peat [56, 57]. The RNA content per gram DW peat did increase from June to August 2018 (Supplementary Fig. S2). This suggests that there might be more metabolically active microbes in the drought period [58]. In drought period, the decreasing water level leads to more oxic conditions and higher oxygen availability in the peat, allowing aerobic microbes to grow better and be more active in nutrient cycling [59]. Thereafter, a decrease of total RNA content was observed from August 2018 to February 2019, indicating less microbial biomass after the drought.

**Figure 4:**
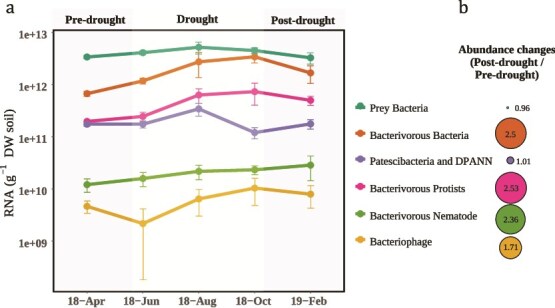
Temporal dynamics of PBac and bacterivore absolute transcript abundances from April 2018 to October 2018 and February 2019, based on metatranscriptomic data. Organisms’ abundances are expressed as SSU rRNA transcripts per gram of dry soil for PBac and bacterivores, and as viral RNA transcripts per gram of dry soil for bacteriophages (a). Plot using a log scale in y axis for visual clarity but show real (original) transcript values as axis labels. Error bars represent standard errors (*n* = 3). The right panel shows the relative changes in transcript abundance of each bacterivorous group before and after the drought (b). Specifically, the transcript abundance in the postdrought month was divided by the abundance in predrought month.

To assess the abundance dynamics of PBac and different bacterivorous groups, the total RNA content was integrated with the relative abundance of RNA transcripts and its molecular mass [39, 40]. The RNA data (Fig. 4a) showed a dominance of PBac, which was in line with the greater Shannon diversity of PBac in the DNA amplicon data (Supplementary Fig. S1). On average, the transcript abundance of PBac was approximately 4.0 × 10^12^ SSU rRNAs per gram of DW soil, with a significant increase from April to August 2018 and a significant decrease from August 2018 to February 2019 (*P* < .05). Similar ribosomal RNA abundances have been reported in other studies of soils [40, 56].

The order of bacterivore transcripts abundance was as follows: BBac > BPro > PD > BNem > bacteriophage (Fig. 4a). The average transcript abundances per gram DW soil were BBac 1.8 × 10^12^, BPro 4 × 10^11^, PD 2 × 10^11^, BNem 2 × 10^10^, bacteriophages 6 × 10^9^, respectively. Similar temporal trends were observed in the BBac, BPro, BNem, and bacteriophages, with a sustained increase of their transcript abundances during the drought period. The transcript abundances of the three aerobic bacterivore group BBac, BPro, BNem more than doubled in the postdrought period compared to predrought (Fig. 4b). Moreover, their transcript abundances showed a peak in October 2018, which came later than the PBac whose abundances peaked in August 2018. Both BBac and BPro transcripts abundance significantly increased from the predrought to the drought period (*P* < .05), followed by a decrease in the postdrought period in February 2019 (Fig. 4a). In contrast, the transcript abundance of PD exhibited a decline at the end of the drought in October, following a similar trend to that of PBac.

**Figure 5:**
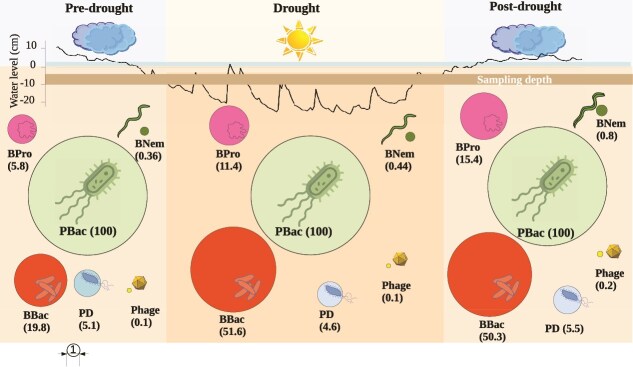
Normalized BPR across pre-, during-, and post-drought periods. BPR was calculated by scaling SSU rRNA bacterivore transcript abundance relative to prey bacterial abundance, which was normalized to 100. PBac: prey bacteria; BBac: bacterivorous bacteria; BPro: bacterivorous protists; PD: *Ca.* Patescibacteria and DPANN. At the top of a figure is a time series graph illustrating changes in water table depth (cm) over times. The size of circles is scaled based on numbers in the brackets.

To consider prey and bacterivore abundance dynamics, we calculated bacterivores-to-PBac transcript ratios (BPR) before, during, and after the drought (Fig. 5) [5]. Remarkably, the BBac- and BPro-to-PBac ratios approximately doubled after the drought onset. For instance, for 100 PBac SSU rRNA transcripts 19.8 BBac transcripts were found before the drought and 51.6 during the drought. In detail, facultative BBac, such as *Myxococcota* (formerly *Myxococcales*), *Cytophagales,* and *Streptomycetaceae* showed similar trends as mentioned above (Supplementary Fig. S3a). In contrast to these facultative BBac, *Lysobacter,* showed a decrease in transcript abundance during the drought (Supplementary Fig. S3a). However, this pattern was associated with high variability among replicates, particularly in August, and therefore should be interpreted cautiously. This observation further indicates that drought responses among facultative predators may be taxon-specific.

Similarly increasing BPR trends during the drought were also observed for two abundant BPro: *Cercozoa* and *Amoebozoa* (Supplementary Fig. S3b). These findings suggest that these aerobic protozoa with predatory strategies would benefit from higher oxygen availability during the drought. This may also be caused by a larger PBac community due to oxic conditions thus increasing their food source. In other words, a higher abundance of PBac might promote the BBac and BPro predation activities, and thus potentially accelerating the nutrient (N, P) cycling in the rewetted fen [60, 61]. Interestingly, obligate BBac (*Vampirovibrionales* and BALOs) exhibited a similar pattern as PBac, with an increase from April to August and a subsequent decrease from August 2018 to February 2019 (Supplementary Fig. S3a). This might indicate the dependence of these obligate bacterivores on their bacterial prey.

The BNem also exhibited an increase in abundance from April 2018 until the postdrought period in February 2019 (Fig. 4a). Correspondingly, the BNem-to-PBac ratio increased as well (Fig. 5). Comparing to the BBac- and BPro-to-PBac ratios, the BNem-to-PBac ratio were increased less during the drought. This may be attributable to the faster generation time of unicellular microorganisms compared to multicellular organisms like nematodes [62, 63]. The structure of the BNem communities underwent a profound and complete change over the drought cycle (Supplementary Fig. S3c). In April 2018, the predominant order was *Araeolaimida*. However, *Triplonchida* exhibited a more rapid increase than *Araeolaimida*, becoming the dominant order during the drought in June 2018. With the severity of the drought, the abundance of *Triplonchida*, *Enoplida*, and *Rhabditida* markedly increased. The *Rhabditida* became the dominant order of BNem in February 2019. These variations may indicate the potential functional shifts of BNem communities in the soil food web due to drought. However, it needs to be considered that our metatranscriptomics approach might have a bias on screening for nematodes since nucleic acid-based methods may not well capture those macroorganisms.

### Bacteriophage transcript dynamics during drought

*Leviviridae,* a family of positive-strand RNA phage, were found in this study, although metatranscriptomics provides limited information on DNA viruses [64–66]. In October 2020, the International Committee on Taxonomy of Viruses (ICTV) renamed the family *Leviviridae* as *Fiersviridae* and reclassified it within the order *Norzivirales* [67]. Despite the lack of a broad insight into the viral community, the *Fiersviridae* found in the peat could be considered an indicator for the general response of bacteriophages and their hosts to the summer drought. In a different study, they also reported that the highest abundances of *Fiersviridae* were found in microcosms with water saturation compared to air-dried soils [68].

*Fiersviridae* target *Proteobacteria* [65], which were the most abundant (approximately 50%) prokaryotes in our data. The fluctuations in the abundance of bacteriophages transcripts (6 × 10^9^ ± 3.6 × 10^9^), which decreased at the onset of drought and the end of the drought (Fig. 4a), may suggest that the bacteriophage are more susceptible to environmental variations [69]. Holistically, the transcript abundance of phages exhibited a 1.7-fold increase in postdrought than in predrought (Fig. 4b). It may suggest the transcriptional activity of RNA bacteriophages also would be triggered by drought. Compared to obligate BBac and PD, the abundance of bacteriophages was not perfectly following the temporal dynamics of PBac (Fig. 4a). This contrasts with our second hypothesis that an abundance of bacteriophages entirely depends on the host bacteria and thus will follow changes in PBac. This indicates that their role in trophic interactions under drought impact still needs further investigation.

Bacteriophage-to-PBac ratios remained stable during the drought (Fig. 4a). The ratio of bacteriophage to the whole PBac is a simplified definition may cause that we do not yet fully understand how bacteriophages interact with their hosts. But the usage of the simplified definition is due to the limited knowledge of phage-host relationships, even we know phages have very narrow host spectrum. We still believe that the incorporation of phages into the trophic interactions is innovative and might pave the way for future, more refined analyses. Additionally, with greater metatranscriptomic non-rRNA gene sequencing depth, the identification of RNA-dependent RNA polymerase sequences, together with phylogenetic analyses, may help to provide a more comprehensive characterization of viral communities [64, 70]. A basis for more accurate predictions of RNA virus–host relationships was provided [71], even though inferring hosts remains challenging.

### Some like it wet: competition drives Patescibacteria and DPANN dynamics?

The SSU rRNA transcripts abundance of *Ca.* Patescibacteria and DPANN archaea (PD) indicated that these small-celled groups were the third most abundant bacterivores in the soil microbiome, with an abundance (2 × 10^11^ transcripts per gram DW soil) that nearly reached that of BPro in April 2018 (Fig. 4a). The high ribosome RNA abundances of these two groups suggests the necessity of considering their roles in the soil food web in rewetted peatlands. The metatranscriptomics approach is especially relevant for the detection of these organisms, because the most commonly used PCR primer pairs for microbiome profiling miss many of these groups.

PD transcript abundances showed a gradual increase between April and August 2018 as water levels declined and the redox potential rose; however, this growth remained slower than that observed in BPro. PD transcript abundances peak in August 2018, likely reflecting transient activity stimulated by host or prey availability. Afterwards (from August to October) their abundance declined as the water level rose closer to the peat surface, which was in line with the decreasing of PBac transcripts abundance and in contrast to the increasing transcript abundances of other bacterivores during the drought period (Fig. 4a). This suggests a strong host dependency, likely reflecting their symbiotic or parasitic associations [25, 27, 30]. This could even indicate their predatory lifestyle as defined in this study (Fig. 1). Accepting the above conclusion, we added PD to the functional guild of bacterivores in the peat. Remarkably, the proportion of PD among the functional guild of bacterivores was rather high predrought (approximately 16% of bacterivore SSU rRNA transcripts; see Fig. 5). Their share dropped drastically during drought, to 6.8% of SSU rRNA transcripts, and remained at 7.6% after the drought. Thus, the observed preference for wet conditions could be a consequence of the lower competitiveness of PD under oxic conditions during the drought, where the obligate aerobic protists, nematodes, Myxobacteria, and BALOs thrive and increase steeply in abundance and consequently comprise the major share of all bacterivores. This may suggest that PD are outcompeted by the aerobic bacterivores, yet remain highly active host cell-associated symbionts/parasites.

Consequently, the following increase in PD transcripts and PD-to-PBac ratio after the drought might suggest that they may compete better with the aerobic bacterivores under wet conditions, probably also due to their parasitic and host-dependent lifestyle [29, 72, 73]. It is worth noting that the Shannon diversity of PD also showed a decreasing trend during the summer drought of 2019 as in 2018. This needs further investigation to understand the impact of repeated droughts on the interaction of PD and their host bacteria.

The most abundant PD, *Ca.* Microgenomates (Supplementary Fig. S4a) and *Ca.* Woesearchaeota (Supplementary Fig. S4b) exhibited a similar pattern to PBac during the drought period. *Ca.* Microgenomates were reported to exhibit both syntrophic/symbiotic and fermentative lifestyles [29, 73]. *Ca.* Parcubacteria were not detected in October 2018 and February 2019, suggesting their low resilience against the drought. The abundance of *CPR2* and *BJ.169* exhibited a decline, which was in contrast to the recovery of *Ca.* Microgenomates in February 2019 (Supplementary Fig. S4a).

### Legacy effects of summer drought

From the drought period to the postdrought period, a shift from oxic to suboxic/anoxic conditions indicated by the redox potential at our sampling depth (Fig. 2b) was observed, with the water level flooding the peat layer. The BBac-to-PBac ratio did not decrease in the postdrought phase, while the BPro- and BNem-to-PBac ratios even increased in the postdrought compared to the drought period (Fig. 5). These results indicate that drought may have a legacy effect on the bacterivores. A doubling in the BBac-, BPro-, and BNem-to-PBac ratios from the predrought to the postdrought month was observed (Fig. 5), indicating that greater postdrought effects resulting from drought may be experienced by aerobic bacterivores. The repeated summer droughts as observed in our study (2018 and 2019, Fig. 2) and in general due to climate change might thus pose an accumulating and profound influence on the ecosystem functioning. There was a consistent increase in the abundance of BNem across the predrought, drought, and postdrought months (Fig. 4a), suggesting that they might continually benefit from drought in the rewetted peatland studied here. This could be attributed to their ability to adapt through physiological changes for surviving environmental extremes [74].

## Conclusion

A unique aspect of this study was the first simultaneous assessment of all bacterivorous groups from the three domains of life, especially the often missed *Ca.* Patescibacteria and DPANN archaea as well as RNA phages. The transcript abundances of aerobic bacterivores and bacteriophages showed an increase during, and a slight decrease after the drought, coinciding with the changes in redox potential that indicate oxic conditions during the drought. Interestingly, none of the bacterivorous groups returned to their predrought abundances and community compositions, rather continuing to increase and shift remained even in the postdrought sample. This indicates a longer-lasting impact of the drought on microbial food web dynamics. With climate change driving more frequent summer droughts, bacterivore responses may cause long-term shifts in ecosystem functioning through altered trophic interactions. This has the potential to accelerate SOC mineralization through the higher abundance of bacterivorous groups with an obligate aerobic energy metabolism, both bacterial and eukaryotic. In turn, these observations hint at an additional factor contributing to C accumulation in anoxic peatlands besides anaerobic metabolism and the enzyme latch mechanism: altered trophic interactions in the microbial food web. A lower abundance of the strictly aerobic bacterivorous groups of nematodes, protists, Myxobacteria and BALOs might result in reduced grazing pressure on saprotrophic bacteria contributing to reduced C mineralization in anoxic peat. Repeated summer droughts could then increase the carbon emissions of rewetted peatlands due to peat oxidation, thereby potentially amplifying climate change. Our findings provide insights on how hydrological disturbance, such as drought, can shift peat microbial food webs and suggest that a stable hydrological regime might be important in securing the carbon through reduced trophic turnover in peatlands, thus mitigating climate change, such as rewetting strategies, can help to stabilize microbial community structure and support the rebuilding of a balanced and healthy peatland ecosystem under climate change.

## Supplementary Material

Supplementary_material_ycag169

## Data Availability

The amplicon sequencing data from April 2017 to February 2018 have been deposited at the European Molecular Biology Laboratory (EMBL) with the project number PRJEB36764 [43]. The 16S rRNA gene, 18S rRNA gene amplicon sequencing and metatranscriptomic sequencing from April 2018 to February 2019 were submitted to the European Nucleotide Archive (ENA) of the EMBL with the project number PRJEB51908 [32]. The amplicon sequencing data from June to October of 2019 were submitted to ENA with the project number PRJEB81734.
