# Supplementary material for: Summer drought impacts micropredator abundance and microbial food web structure in a rewetted fen peatland
Source: ISME Commun. 2026 Jun 16;6(1):ycag169. doi: 10.1093/ismeco/ycag169 (PMC13477217; doi:10.1093/ismeco/ycag169)
Supplement: Supplementary file 1 [file supplementary_material_ycag169.zip › Supplementary_methods_figures_ycag169.pdf]

Supplementary information:

Summer drought impacts micropredator abundance and microbial foodweb structure in a rewetted fen peatland

Short Title: drought impacts micropredator abundance

Authors: Die Hu[1], Haitao Wang[1,2*], Verena Groß[1], Anna M. Burns[1], Tim Urich[1]

[1]Institute of Microbiology, University of Greifswald, 17489 Greifswald, Germany

[2]Grassland and Forage Sciences, University of Rostock, 18059 Rostock, Germany

Corresponding author: Haitao Wang haitao.wang@uni-greifswald.de

This supplementary material includes:

Supplementary methods

Supplementary figures S1 to S4

Supplementary methods

Peat soil sampling and processing

The percolation fen is located in the Trebel and Recknitz river valleys of Mecklenburg-Vorpommern in Northeastern Germany and has been rewetted by groundwater from river catchment over the past two decades [1]. The soil samples from the rewetted fen were collected from WETSCAPES project sites [2]. Further details regarding the rewetted fen, including its location, peat characteristics, and management, have been presented by Jurasinski et al., [2]. The samples were collected in triplicate from April 2017 to October 2019 (April, August, and November of 2017, February, April, June, August, October, December of 2018, and February, June and, October of 2019) at a depth of 5-10 cm below the soil surface. The soil samples were thoroughly mixed and transported to the laboratory on ice (for DNA extraction) or dry ice (for RNA extraction) and were stored at −20 °C or −70 °C respectively until further processing. Further details regarding the sampling methods can be found in previous publications [3–5]. A total of 36 samples were collected (12 time points × 3 replicates) for amplicon sequencing. Soil parameters including water level, redox potential, pH, soil moisture, salinity, and other nutrients, were measured as detailed in Weil et al. [6]. Measurement of the water level began in September 2017 and continued through the end of the study period in October 2019. To further capture all the above-mentioned organisms and compare their response to drought using a single method, total RNA metatranscriptomic sequencing was conducted for samples from April to October 2018 and in February 2019. The drought period, which occurred from June to October 2018, was characterized by a decline in water level that remained below the sampling depth (5-10 cm). In total, 15 samples were collected (5 time points × 3 replicates) for metatranscriptomic sequencing.

DNA and RNA extraction and sequencing

Soil DNA was extracted using the DNeasy PowerSoil Kit (QIAGEN, Hilden, Germany), following the manufacturer's instructions with some modification. For bead beating, a FastPrepR -24 5G instrument (MP Biomedicals, Santa Ana, USA) with an intensity of 5.0 m/s for 45 seconds was used. The extracted DNA samples were sent to LGC Genomics GmbH (Berlin, Germany) for sequencing. The 16S rRNA and 18S rRNA gene amplicons were sequenced using the Illumina MiSeq 300 bp paired-end platform as previously described [3, 4].

The total RNA was extracted using the RNeasy PowerSoil Total RNA Kit (QIAGEN, Hilden, Germany). The extracted RNA was subsequently treated with DNase I and purified using the MEGAclear Kit (Thermo Fisher Scientific, Waltham, MA). The mRNA fraction was enriched using the MessageAmp™ II-Bacteria RNA Amplification Kit (Thermo Fisher Scientific, MA). The amplified RNA was used for the sequencing library preparation with NEBNext Ultra II RNA Library Prep Kit for Illumina (New England Biolabs, Ipswich, MA). The metatranscriptomic libraries were sequenced using an Illumina NextSeq 500/550 system. Further details regarding the sequencing can be found in previous publications [4, 5].

Bioinformatics

All raw amplicon sequence data were demultiplexed with barcodes, adapters, and primers removed using the Illumina bcl2fastq software and then processed using the dada2 (v1.8.0) pipeline [3]. The final representative sequence of each amplicon sequence variant (ASV) was assigned to taxonomy against a modified version of the SILVA SSU ref NR 128 database using the lowest common ancestor (LCA) algorithm with MEGAN5 [7]. ASVs assigned to chloroplasts or mitochondria based on 16S rRNA gene amplicon data were excluded from the analysis. Furthermore, ASVs detected in only one single sample were excluded in order to reduce the potential for sample-specific outlier effects. Poten-

tial bacterivores were assigned to four categories: BBac, CPR and DPANN archaea from 16S rRNA, as well BNem and BPro from 18S rRNA (Table 1). Most of the CPR bacteria have been reassigned to the superphylum *Ca.* Patescibacteria in the SILVA 138 SSU database [8]. In the following text, the *Ca.* Patescibacteria and DPANN archaea are referred to as PD. Bacterial groups that did not belong to any of the predatory bacterial groups were defined as non-predatory prey bacteria (PBac) [9].

The processing of metatranscriptomic data was described in Wang et al., [4]. Briefly, the forward and reverse raw sequences were merged using FLASH [10] and trimmed using PrinseqLite [11]. SortMeRNA (v2.1) was then used to categorize the sequences into small subunit (SSU) rRNA, large subunit (LSU) rRNA and non-rRNA (putative mRNA) fractions [12]. SSU rRNA sequences (230-250 bp) were subsampled to 200,000 per sample using USEARCH [13]. To maintain consistency throughout the study, taxonomy was assigned with the same SILVA 128 database using the LCA algorithm (Min Score 155; Max Expected 0.01; Top Percent 4.0; Min Support 1) with MEGAN6 [14]. However, the latest taxonomic names from the new database have adapted when necessary, e.g., *Ca.* Patescibacteria (which was CPR in the old database). Putative mRNA reads were aligned to protein sequences in the NC_nr database (accessed 12/04/2020) using DIAMOND [15], with taxonomies assigned in MEGAN5 [7] via the LCA algorithm. On average, 3,040 reads per sample corresponded to viral sequences, of which 42.7% were classified to bacteriophages from the family *Leviviridae*. which may infect *Proteobacteria* [16], were considered as bacterivores for downstream analysis. In October 2020, the International Committee on Taxonomy of Viruses (ICTV) renamed the family *Leviviridae* as *Fiersviridae* and reclassified it within the order *Norzivirales* [17]. A total of 14 metatranscriptomic samples remained for the downstream analyses. One of 15 samples was excluded because it diverged strongly from the other two replicates, which may be due to contamination during sample processing.

Statistics analyses

R v4.1.2 was employed for the statistical analyses (http://www.r-project.org/). For amplicon sequencing data, the Shannon index was utilized to estimate the community richness and evenness using the *phyloseq* (v1.50.0) package [18]. A non-parametric Kruskal-Wallis test and *post-hoc* Dunn test for pairwise comparisons were conducted to test the differences in soil properties or bacterivores between years or seasons using the *rstatix* (v0.7.2) package [19]. After normalizing bacterivore ASV counts with the Hellinger transformation, community composition was analyzed via Principal Coordinates Analysis (PCoA) based on Bray-Curtis distances. Permutational multivariate analysis of variance (PERMANOVA) was performed to test the effects of different time points on bacterivores community composition employing the *adonis2* function of *vegan* package [20]. The correlations between abundance of bacterivores and the abundance of prey bacteria and other edaphic properties were tested using *cor.test* function in the *stats* (v4.4.2) package with Spearman correlation. All p-values for multiple comparisons were adjusted by the FDR method and the null hypothesis was rejected for adjusted $p < 0.05$. All figures were implemented in *ggplot2* (v3.5.2) package [21]. The absolute abundance of SSU rRNA transcripts and viral RNA was calculated based on total RNA content [5, 22, 23]. In briefly, the theoretical ratio of mRNA: SSU rRNA : LSU rRNA is 4:32:64 for the total RNA per sample. The length of mRNA, 16S and 18S SSU rRNA were about 1,000, 1,500 and 1,900 nucleotides, respectively. With the Avogadro constant of ca. 6.02×10^{23} units per mol, we estimated mRNA, 16S and 18S rRNA transcripts per gram dry weight (DW) soil. Bacteriophage abundance was calculated according to the classified mRNA putative reads obtained by filtering out SSU and LSU rRNA [22]. The calculation of the bacterivore-to-prey bacteria ratio involved the ratio of transcript abundance of bacterivorous groups to that of prey bacteria, scaled to 100, based on RNA transcripts per gram of DW soil.

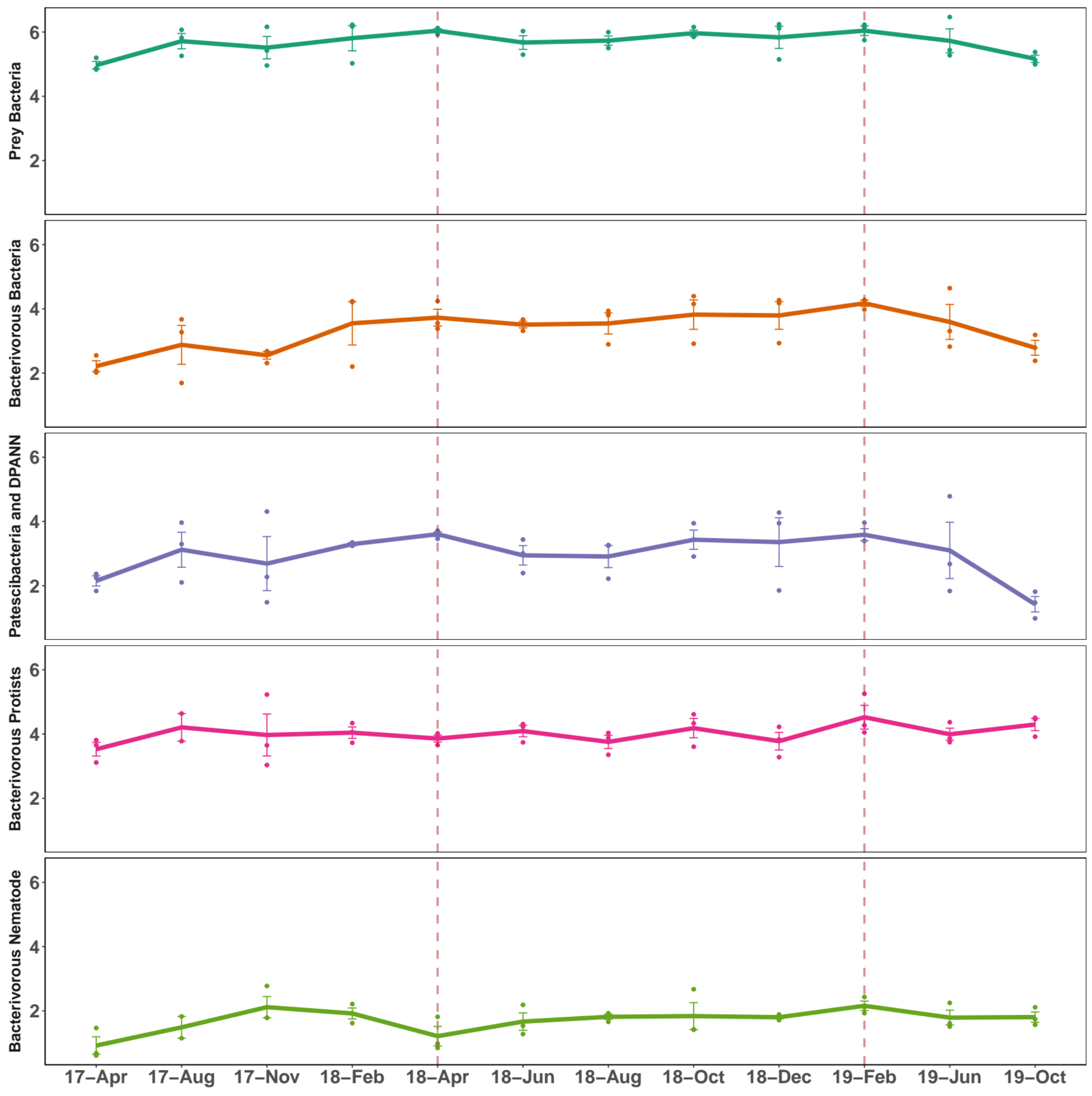

Supplementary Figure S1. **The Shannon index of prey bacteria and bacterivores from the DNA-based amplicon sequencing data.** The dashed vertical lines indicate the periods for metatranscriptomics analysis with the exception of 18-Dec. All values are averaged and represented with their standard error (n = 3)

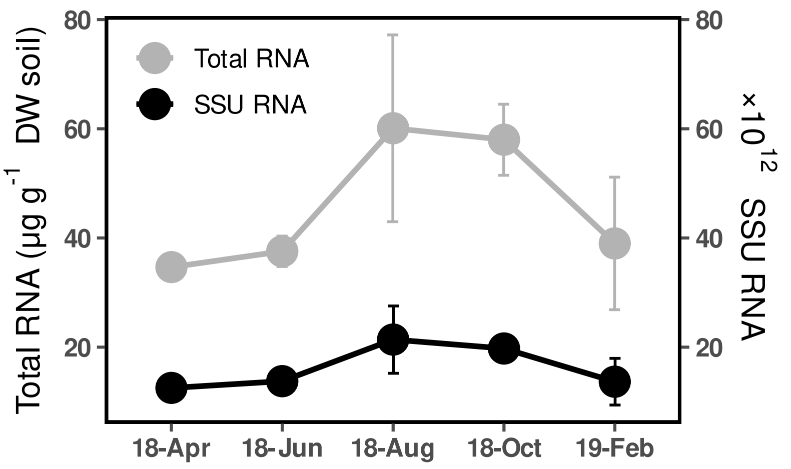

Supplementary Figure S2. **Concentration of total extracted RNA (µg/g DW soil) and the absolute transcript abundance of SSU rRNA per gram soil (dry weight, DW) quantified from the metatranscriptomics sequencing data (Söllinger et al., 2018).** All values are averaged and represented with their standard error (n = 3).

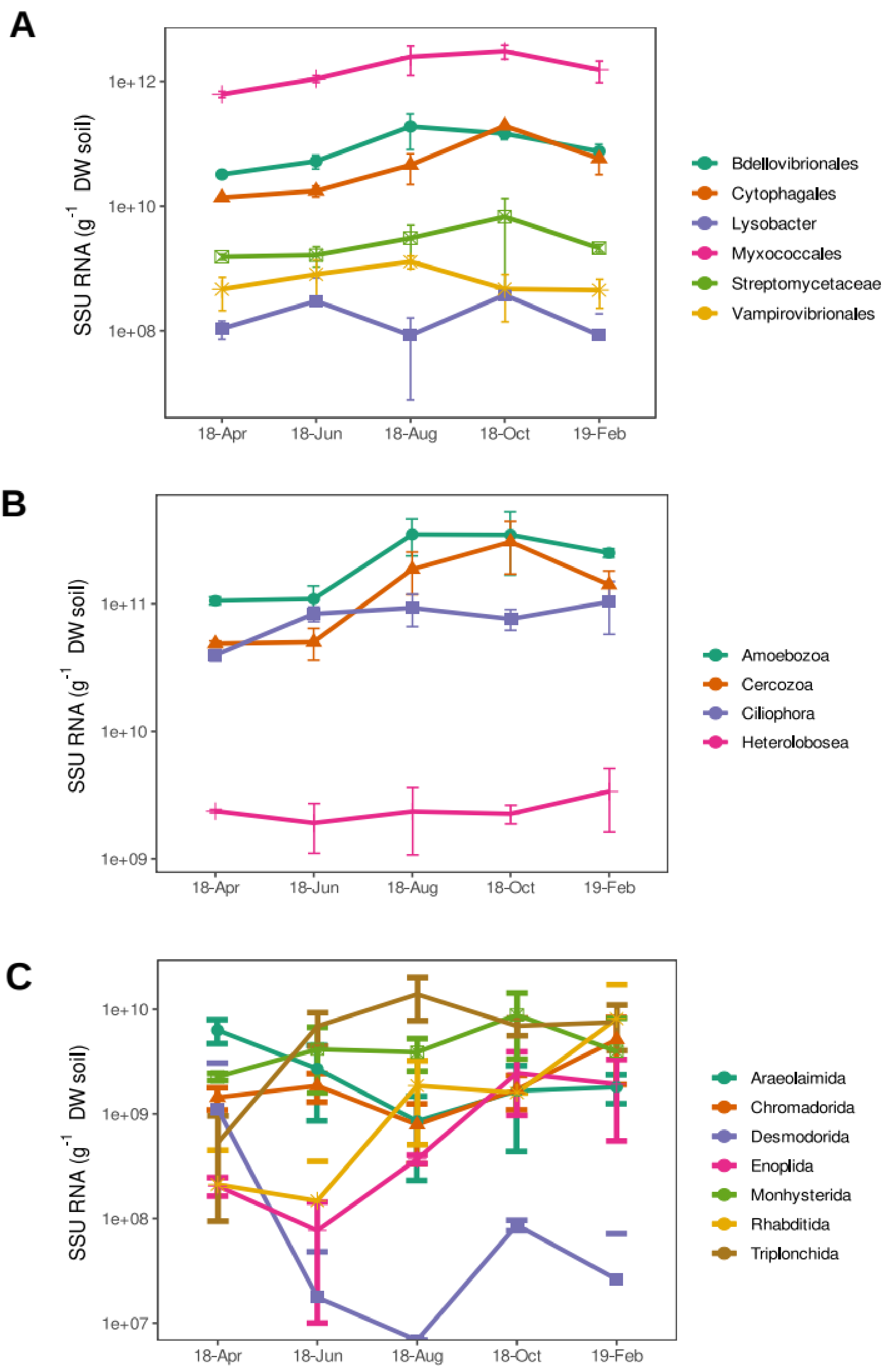
A
SSU RNA (g-1 DW soil)
1e+12
1e+10
1e+08
18-Apr
18-Jun
18-Aug
18-Oct
19-Feb
Bdellovibrionales
Cytophagales
Lysobacter
Myxococcales
Streptomycetaceae
Vampirovibrionales
B
SSU RNA (g-1 DW soil)
1e+11
1e+10
1e+09
18-Apr
18-Jun
18-Aug
18-Oct
19-Feb
Amoebozoa
Cercozoa
Ciliophora
Heterolobosea
C
SSU RNA (g-1 DW soil)
1e+10
1e+09
1e+08
1e+07
18-Apr
18-Jun
18-Aug
18-Oct
19-Feb
Araeolaimida
Chromadorida
Desmodorida
Enoplida
Monhysterida
Rhabditida
Triplonchida

Supplementary Figure S3. **Temporal dynamics of the absolute abundance of SSU rRNA per gram of dry weight soil of bacterivorous bacteria (a), phylum level of bacterivorous protists (b), and order level of bacterivorous nematodes (c).** Taxonomy classification was assigned using the SILVA 128 database which was the old database. Plot using a log scale in y axis for visual clarity, but show real (original) transcript values as axis labels.

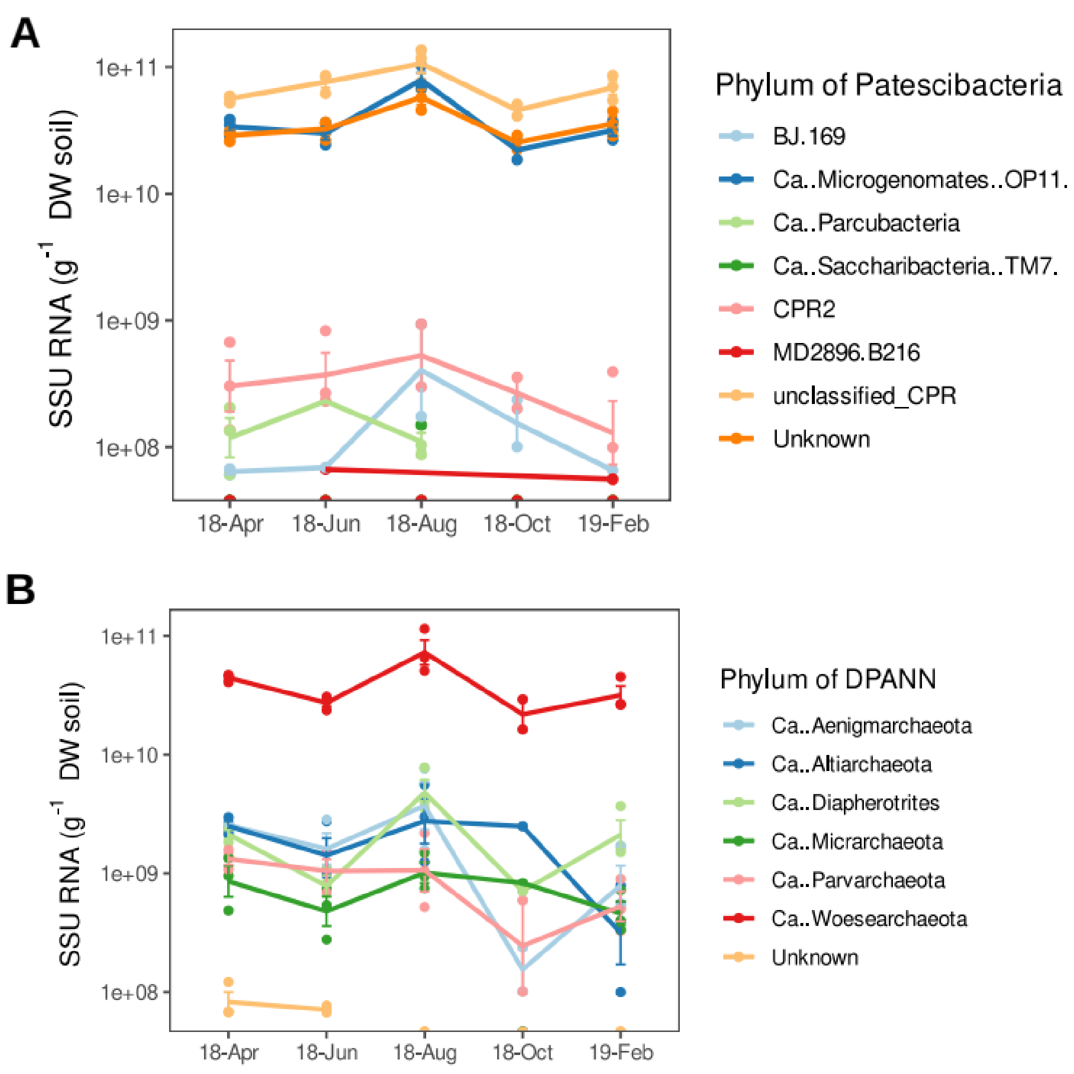

Supplementary Figure S4. **Seasonal/annual/temporal dynamics of the absolute abundance of SSU rRNA per gram of dry weight soil of Ca. Patescibacteria (A) and DPANN (B).** Taxonomy classification was assigned using the SILVA 128 database. Plot using a log scale in y axis for visual clarity, but show real (original) transcript values as axis labels.

Reference

1. Zerbe S et al. Ecosystem Service Restoration after 10 Years of Rewetting Peatlands in NE Germany. *Environ Manage* 2013;**51**:1194–1209. https://doi.org/10.1007/s00267-013-0048-2
2. Jurasinski G et al. From Understanding to Sustainable Use of Peatlands: The WETSCAPES Approach. *Soil Syst* 2020;**4**:14. https://doi.org/10.3390/soilsystems4010014
3. Wang H et al. Eukaryotic rather than prokaryotic microbiomes change over seasons in rewetted fen peatlands. *FEMS Microbiol Ecol* 2021;**97**:fiab121. https://doi.org/10.1093/femsec/fiab121
4. Wang H et al. The impact of summer drought on peat soil microbiome structure and function-A multi-proxy-comparison. *ISME Commun* 2022;**2**:1–4. https://doi.org/10.1038/s43705-022-00164-x
5. Wang H et al. Linking Transcriptional Dynamics of Peat Microbiomes to Methane Fluxes during a Summer Drought in Two Rewetted Fens. *Environ Sci Technol* 2023;**57**:5089–5101. https://doi.org/10.1021/acs.est.2c07461
6. Weil M et al. Long-Term Rewetting of Three Formerly Drained Peatlands Drives Congruent Compositional Changes in Pro- and Eukaryotic Soil Microbiomes through Environmental Filtering. *Microorganisms* 2020;**8**:550. https://doi.org/10.3390/microorganisms8040550
7. Huson DH et al. Integrative analysis of environmental sequences using MEGAN4. *Genome Res* 2011;**21**:1552–1560. https://doi.org/10.1101/gr.120618.111
8. Glöckner FO et al. 25 years of serving the community with ribosomal RNA gene reference databases and tools. *J Biotechnol* 2017;**261**:169–176. https://doi.org/10.1016/j.jbiotec.2017.06.1198
9. Petters S et al. The soil microbial food web revisited: Predatory myxobacteria as keystone taxa? *ISME J* 2021;**15**:2665–2675. https://doi.org/10.1038/s41396-021-00958-2
10. Magoč T, Salzberg SL. FLASH: fast length adjustment of short reads to improve genome assemblies. *Bioinformatics* 2011;**27**:2957–2963. https://doi.org/10.1093/bioinformatics/btr507
11. Schmieder R, Edwards R. Quality control and preprocessing of metagenomic datasets. *Bioinformatics* 2011;**27**:863–864. https://doi.org/10.1093/bioinformatics/btr026

12. Kopylova E, Noé L, Touzet H. SortMeRNA: fast and accurate filtering of ribosomal RNAs in metatranscriptomic data. *Bioinformatics* 2012;**28**:3211–3217. https://doi.org/10.1093/bioinformatics/bts611
13. Edgar RC. Search and clustering orders of magnitude faster than BLAST. *Bioinformatics* 2010;**26**:2460–2461. https://doi.org/10.1093/bioinformatics/btq461
14. Huson DH et al. MEGAN Community Edition - Interactive Exploration and Analysis of Large-Scale Microbiome Sequencing Data. *PLOS Comput Biol* 2016;**12**:e1004957. https://doi.org/10.1371/journal.pcbi.1004957
15. Buchfink B, Xie C, Huson DH. Fast and sensitive protein alignment using DIAMOND. *Nat Methods* 2015;**12**:59–60. https://doi.org/10.1038/nmeth.3176
16. Starr EP et al. Metatranscriptomic reconstruction reveals RNA viruses with the potential to shape carbon cycling in soil. *Proc Natl Acad Sci* 2019;**116**:25900–25908. https://doi.org/10.1073/pnas.1908291116
17. Krupovic M et al. Bacterial Viruses Subcommittee and Archaeal Viruses Subcommittee of the ICTV: update of taxonomy changes in 2021. *Arch Virol* 2021;**166**:3239–3244. https://doi.org/10.1007/s00705-021-05205-9
18. McMurdie PJ, Holmes S. phyloseq: An R Package for Reproducible Interactive Analysis and Graphics of Microbiome Census Data. *PLOS ONE* 2013;**8**:e61217. https://doi.org/10.1371/journal.pone.0061217
19. Kassambara A. rstatix: Pipe-Friendly Framework for Basic Statistical Tests. 2023. 2023.
20. Oksanen J et al. vegan: Community Ecology Package. 2024. 2024.
21. Wickham H et al. ggplot2: Create Elegant Data Visualisations Using the Grammar of Graphics. 2024. 2024.
22. Söllinger A et al. Holistic Assessment of Rumen Microbiome Dynamics through Quantitative Metatranscriptomics Reveals Multifunctional Redundancy during Key Steps of Anaerobic Feed Degradation. *mSystems* 2018;**3**:10.1128/msystems.00038-18. https://doi.org/10.1128/msystems.00038-18

23. Täumer J et al. Linking transcriptional dynamics of CH_4-cycling grassland soil microbiomes to seasonal gas fluxes. *ISME J* 2022;**16**:1788–1797. https://doi.org/10.1038/s41396-022-01229-4
